# Parasite Polymorphism and Severe Malaria in Dakar (Senegal): A West African Urban Area

**DOI:** 10.1371/journal.pone.0009817

**Published:** 2010-03-23

**Authors:** Ndeye Sakha Bob, Bernard Marcel Diop, Francois Renaud, Laurence Marrama, Patrick Durand, Adama Tall, Boubacar Ka, Marie Therese Ekala, Christiane Bouchier, Odile Mercereau-Puijalon, Ronan Jambou

**Affiliations:** 1 Institut Pasteur de Dakar, Dakar, Senegal; 2 CHU Fann, Clinique des maladies infectieuses, Dakar, Senegal; 3 U.M.R. 2724 CNRS-IRD G.E.M.I., Montpellier, France; 4 Hôpital Principal de Dakar, Dakar, Senegal; 5 Institut Pasteur, Immunologie Moléculaire des Parasites, CNRS URA 2581, Paris, France; 6 Institut Pasteur, Genopole de l'Ile de France, PF1, Paris, France; Université Pierre et Marie Curie, France

## Abstract

**Background:**

Transmission of malaria in West African urban areas is low and healthcare facilities are well organized. However, malaria mortality remains high. We conducted a survey in Dakar with the general objective to establish who died from severe malaria (SM) in urban areas (particularly looking at the age-groups) and to compare parasite isolates associated with mild or severe malaria.

**Methodology/Principal Findings:**

The current study included mild- (MM) and severe malaria (SM) cases, treated in dispensaries (n = 2977) and hospitals (n = 104), We analysed *Pfdhfr*/*Pfcrt*-exon2 and nine microsatellite loci in 102 matched cases of SM and MM. Half of the malaria cases recorded at the dispensaries and 87% of SM cases referred to hospitals, occurred in adults, although adults only accounted for 26% of all dispensary consultations. This suggests that, in urban settings, whatever the reason for this adult over-representation, health-workers are forced to take care of increasing numbers of malaria cases among adults. Inappropriate self treatment and mutations in genes associated with drug resistance were found associated with SM in adults. SM was also associated with a specific pool of isolates highly polymorphic and different from those associated with MM.

**Conclusion:**

In this urban setting, adults currently represent one of the major groups of patients attending dispensaries for malaria treatment. For these patients, despite the low level of transmission, SM was associated with a specific and highly polymorphic pool of parasites which may have been selected by inappropriate treatment.

## Introduction

The UNESCO estimates that half of the African population will be living in an urban setting by 2030 [1]. This urbanization is associated with better access to healthcare. However, the fatality rate of malaria is not decreasing in these urban settings and it is hence urgent to better understand the factors contributing to mortality.

Urbanization profoundly changes the epidemiology of malaria, causing a decrease in risk, when compared to rural areas [1]–[3]. The Dakar area, with one-fifth of the Senegalese population, is a good example of this new setting, as it undergoes rapid urbanization and combines urban and peri-urban farming zones. Malaria transmission used to occur near natural surface water collections (“niayes”), but a combination of drought (occurring during the last 30 years) and urbanization dramatic decreased this way of transmission. The parasitic index in children has fallen from 80% in 1967, to less than 10% in the 90's [4], with only 10.6% of febrile illnesses due to malaria [5]. Since 1992, transmission of malaria is low and limited to the rainy season [6]. The parasitological index has fallen to less than 2.4% with no difference between adults and children [7]–[8]. In peri-urban areas, most patients visit numerous dispensaries and those with severe malaria are referred to hospitals. Chloroquine and sulfadoxine/pyrimethamine are still commonly used as self-medication instead of artesunate-containing therapies (ACT). The statistics provided by suppliers confirm a large consumption of antimalarial drugs in this area inducing substantial drug pressure favouring the selection of drug-resistant parasites or of a selection of a specific group of more effective (virulent?) parasites which can contribute to mortality [9]–[12].

To better document severe malaria cases in this urban setting, we conducted a survey in one of the biggest dispensaries and in two main hospitals. In this epidemiological setting, young adults were the major group at risk from malaria and severe malaria (SM) in both, dispensaries and hospitals. Self treatment and a delay in consultation appeared to be the major factors associated with SM. However, genetic analyses of the parasite isolates from SM and MM, using molecular markers, of drug resistance to chloroquine and pyrimethamine as well as parasite polymorphic (neutral microsatellite loci, [13]) revealed a very large parasite diversity in the isolates. It also confirmed clear genetic differences within parasites associated with MM.

## Methods

### Recruitment of patients

In Senegal, mild malaria cases are managed at the dispensary and severe malaria cases are referred to a hospital. Patients with mild malaria, were registered during the rainy season (from September to December) at the Dispensaire St-Martin in Dakar. Routine registration and questioning of patients, medical examination, biological diagnosis and treatment were conducted by eight nurses and two medical doctors, in agreement with the Senegalese national recommendations. Medical consultations of adults, children and pregnant women were conducted in separate areas. All patients with fever and without obvious etiology underwent a thick blood smear examination prior to antimalarial treatment. Mild malaria was defined as fever associated with a positive thick smear and without other obvious causes of fever. Severe malaria cases were assessed by the local team, according to the national recommendation (mainly based on clinical criteria) and referred to a hospital. All biological examinations were confirmed at the Institut Pasteur de Dakar. Malaria cases were recruited by our team from two hospitals in Dakar, namely *Centre hospitalier national* (CHN) de Fann, and Hôpital Principal de Dakar (HPD), over the same period of time. In the hospitals, patients with fever and clinical signs of cerebral impairment underwent a lumbar puncture (to rule out meningitis) and twice rapid malaria diagnosis tests plus a thick blood smear, over a 48 hour period. The same protocol of examination and questioning (of the family) was followed as for MM. Assignment of patients to a cerebral malaria (CM) group was carried out following the WHO criteria. However, *P.falciparum*-positive patients with impaired consciousness but not fulfilling WHO definition, were called “hospitalized malaria” (HM). Most of them experienced a rapid recovery of consciousness during the time of presentation. During statistical analysis when no difference was found between the CM and HM groups, patients of the two groups were pooled into the severe malaria (SM) group for further analysis.

During this survey patients with MM were matched with SM for the genotyping study. This group of MM matched patients was submitted again to a careful interrogation (especially about drug use and self-medication) and physical examination. Immediately at presentation of a patient with SM, two MM cases were matched based on their area of residence, age group and gender. 144 MM were matched before the assignment of hospitalized patients to CM or HM groups. Only 102 control patients were kept as matched controls for the genotyping study after confirmation of the MM and SM status (table 1).

**Table 1:** Characteristics of the patients recruited for the parasite genotyping study.

	Cerebral malaria			Hospitalized malaria			Mild malaria			Total
	F	M	Total	F	M	Total	F	M	Total	
**Number of patients (M/F ratio)**	**16**	**18**	**34 (1.12)**	**16**	**25**	**42 (1.56)**	**56**	**46**	**102 (0.82)**	**178**
**Mean age (SD)**	12.5	16.2	14.5 (10.3)	19	13.3	15.5 (13)	16.5	10.3	13.7 (11)	14.3
**Patients treated before consultation (%)**	43.8%	33.3%	38.2%	43.8%	60%	52.4%	12.5%	22.2%	16.8%	29.4%
**Use of prophylaxis (% of patients)**	18.8%	16.7%	**17.6%**	18.8%	24%	**21.4%**	12.5%	13.3%	**13%**	15.8%
**Patients with a temperature >40°C (%)**	6.2%	22.2%	14.7%	12.5%	12%	11.9%	14.3%	13.3%	14%	13.6%
**Percent of deaths**	19%	22%	21%	0	0	0	0	0	0	-
**Parasitaemia** * **(paras./µL)**	5165	1297	2656	756	1736	1276	15943	6343	10436	5370
**Hemoglobin (mean g/L)**	8.3	10.7	9.7	-	-	-	10	10%	10.6	-
**Prevalence of anemia** **	66.7%	50%	57%	-	-	-	33.3%	32.5%	33%	36%
**Mean delay before consultation (days)**	5.1	4.8	4.9	5	7.3	6.4	3.3	3.7	3.45	4.4
**Patients consulting >4 days after onset of the symptoms (%)**	53.3%	58.8%	56.2%	46.7%	60%	55%	16.7%	29.5%	22.5%	36.5%

*Parasitaemia was counted on Giemsa stained thick smears for 1000 leukocytes and expressed as parasites per microliter of blood, according to WHO standards.

Geometric means were calculated.

**Haemoglobin less than 10 g/dL.

Parasiteamia was measured by examination of Giemsa stained blood smears using a magnification ×1000 and by establishing the parasite count per 1000 leukocytes. The count per microliter was calculated according to WHO recommendations.

For each patient enrolled in the genotyping study, 5 mL of peripheral venous blood was collected on EDTA.

Written consent was obtained from the patients, or their relatives, after verbal information in the vernacular language, and the study was approved by the National Ethics Committee of the Ministry of Health of Senegal.

### DNA extraction and analysis

Parasitic DNA was extracted using the standard Phenol-Chloroform procedure [14]. *Pfcrt* exon_2, and *Pfdhfr* were sequenced as described [15]. Nine single-copy microsatellite sequences (BM27, PFPK2, TAA109, TAA87, 2490, ARA2, PFG377, 7A11, TAA42) [13], [16] were amplified using semi-nested PCR (Table 2). PCR products were analyzed, in a random order in single blind experiments, on an ABIprism_377 DNA sequencer.

**Table 2:** Microsatellites sequenced and primers used for their genotyping.

	chromosome	size (bp)	Accession	PCR 1		PCR 2		
				Primers	Ta* (°C)	Primers	Ta *(°C)	
**BM27**	Chr9	133	G44484	CAAAAGAAGTAATATATGTGC TGACTCTTTGATTATATACC	42	TGACTCTTTGATTATATACC CAAAAGAAGTAATATATGTGCCC		42
**7A11**	Chr7	112	G38831	ATGTGTAAGGAGATAGTATA GTAATATTTAAAAAGAGAAG	42	GTAATATTTAAAAAGAGAAG TTTGTAATATTTAAAAAGAGAAG		42
**TAA42**	Chr5	183	G38832	ACAAAAGGGTGGTGATTCT CTTTAGTAGTAGTAATAATAC	42	CTTTAGTAGTAGTAATAATAC TAGAAACAGGAATGATACG		42
**2490**	Chr10	87	G37790	TTCTAAATAGATCCAAAGATG ATGTGCAGATGACGA	44	ATGATGTGCAGATGACGA GTGCATTCAATAATTCTA		44
**ARA2**	Chr11	70	G37848	GTACATATGAATCACCAA TATTAATAATACTCAAAGC	42	TATTAATAATACTCAAAGC GAATAAACAAAGTATTGCT		42
**PFGG377**	Chr12	98	G37851	GATCTCAACGGAAATTAT TTATGTTGGTACCGTGTA	42	TTATGTTGGTACCGTGTA TGTTAATCGTAGGGATAA		42
**PFPK2**	Chr12	168	G37852	CTTTCATCGATACTACGA ATCATGCATTTCAGTCTGAGG	42	ATCATGCATTTCAGTCTGAGG TCTGCTTGTTCCTTCTTT		42
**TAA87**	Chr6	112	G38838	ATGGGTTAAATGAGGTACA GAGTAATA TGAACATGT	42	GAGTAATA TGAACATGT AATGGCAACACCATTCAAC		42
**TAA109**	Chr6	161	G438842	TAGGGAACATCATAAGGA TAGCATGTTTGGTATAGG	42	TAGCATGTTTGGTATAGG GGTTAAATCAGGACAACAT		42

*Ta  =  annealing temperature.

For all microsatellites, PCR extension was carried out at 62°C for 40 sec.

### Population genetic structure and linkage disequilibrium (LD)

Differences between groups were compared using the Mann-Whitney (MW), Chi2 or Kruskal-Wallis (KW) tests. Correlations were calculated using a Spearman test (SP).

Polymorphism of the parasite populations was determined by the complexity of the isolates, i.e. the average number of parasite genotypes per patient, in the population (estimated for each patient from the locus displaying the largest number of alleles) and for each microsatellite by i) the mean number of alleles per patient and ii) the number of alleles needed to describe 50% or 75% of the patients in the group. For population genetic analyses, individual haplotypes were reconstructed for the nine microsatellites, when possible (single allele observed at each locus or multiple alleles observed at only one locus). Genetic variability was quantified using FSTAT as i) mean number of alleles (*A*), and ii) expected heterozygosity under panmixia (*H*_E_) [17]. Linkage disequilibrium was tested by the permutation procedure, using FSTAT for the whole population or for each of the three populations (i.e. Fann, HPD and dispensary) separately and by canonical analysis [18].

## Results

### Mild malaria cases in dispensaries

**Figure 1:**
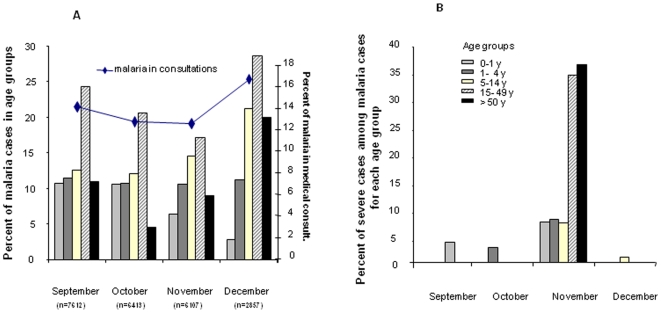
Recruitment of mild malaria cases in dispensaries between September and December (2004). Consultations are cumulated monthly (n). Patients of 5–14 y, 15–49 y and ≥50 y represented 19%, 26.5% and 8.4% of the medical consultations. A) Monthly prevalence of malaria in total medical consultations and prevalence in each age group. Malaria infection increased from October-November (12.6%) to December (16.7%, Chi2, p = 0.000014) in medical consultations, especially for older patients (from 13% to 21% for 5–15 y patients, Chi2 p = 0.000004; from 24% to 28.5% for 15–49 y, Chi2 p = 0.018) but not for infants (decrease from 10.7% to 2.7%, Chi2 p<0.0000001). B) Percentage of severe malaria in malaria cases, per month and per age group,. and representation of each age group in these severe cases.

During this study 22,989 patients attended the *Dispensaire St Martin* for medical consultations, with a decrease from September to December (7,612 to 2,685). Overall, 46% of the consultations were for children less than 5 years old (21% for infants <1 y, 25% for 1–5 y olds). Older patients (>15 y) represented 26% of the consultations and their number increased during November and December. Malaria attacks accounted for 13.7% of all the consultations (n = 2977) (Fig. 1A) and increased from October to December, especially for older patients (Fig. 1A). Infants less than 5 years old accounted for 17.8% of all malaria cases in September, but only 3.3% in December. Adults (>15 y) represented more than half of all malaria cases registered during the study (41% and 61% of all malaria cases in September-October and November-December, respectively), which represents a 2-fold higher rate for the age-group of 15–45year olds as compared to children (OR = 1.84 [1.7–1.98]). For the age-group of 14–49year olds a significant difference was found during consultation in the male/female ratio, as women accounted for 65% of the consultations. Likewise, in this age-group the prevalence of malaria was higher among women (23% of the consultations versus 19% for men, OR = 1.35 [1.1–1.54]). Under the age of 1 year, boys were more often referred to dispensaries for malaria than girls (11% of consultations versus 6% for girls OR = 1.77 [1.43–2.19]).

Cases referred by the dispensary to hospitals were more frequent in November (24% of the malaria cases) than in any other month (average 5%, Fig. 1B). Patients over 15years represented 87% of those referred cases, with an over-representation of females (10.2% of SM among females attending dispensary with malaria versus 5% of SM among males malaria cases, OR = 2.15 [1.62–2.85]), especially for patients more than 15 years old (18.3% of women versus 11.7% of men, OR = 1.62 [1.22–2.33]). During this study only *P.falciparum* was reported.

### Treatment delay and self-treatment are more frequently associated with severe malaria patients

Overall 29.4% of the patients enrolled in this study (MM+CM+HM) used self-treatment before consultation, with a significant difference between groups (Tab. 1; MM versus HM CHI^2^ p = 0.0001, MM vs. CM CHI^2^ p = 0.009), but not in the use of prophylaxis (16%) (not significant, NS). Severe cases (CM+HM) had experienced a longer delay before consultation than MM cases (CHI2 p = 0.0005), especially among the patients using self-treatment (Chi2 p = 0.00015). Geometric mean parasitaemia was higher in MM than in severe cases (Tab. 1) and was lower in patients who underwent self-treatment (2,343 with self-treatment versus 7,499 parasites/µL without, t-student p<0.00001), suggesting partial efficacy of the drugs being responsible for the delayed consultation. No correlation was found between age and parasitaemia levels, reflecting prior drug consumption or lack of immunity. Anaemia was more frequent in CM than in MM cases and concerned females more often than males (MW p = 0.05) or younger patients (correlation haemoglobin vs age, Spearman R = 0.2520, p = 0.0076).

### High rate of *Pfdhfr* mutations in severe malaria patients

**Figure 2:**
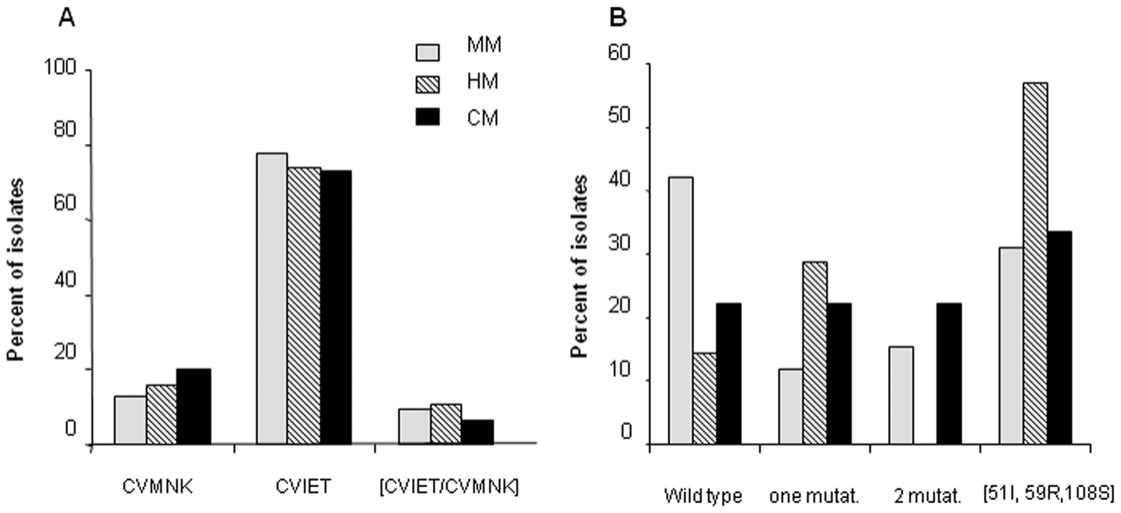
Mutations in *Pfcrt* and *Pfdhfr* genes. A) Pfcrt-exon-2 haplotypes. 110 sequences were obtained (n = 15 CM, 19 HM, 76 MM). Only two haplotypes were detected in codons 72–76, CVNMK (wild type, n = 16) and CVIET (mutated, n = 84). B) Frequency of distribution of *Pfdhfr* alleles (n = 43, 9 CM, 7 HM and 26 MM). Three mutations were found: N51I, C59R, and N108S.

Due to the small quantity of blood sampled from each patient, only 110 samples were sequenced for *Pfcrt*-exon2 (Fig. 2A). Only codons 74, 75 and 76 were found to have mutated, with two haplotypes identified (CVMNK or CVIET) and ambiguous sequences consistent with the presence of both haplotypes (Fig. 2A). No difference was found in the distribution of these genotypes in MM, CM or HM samples. Mutations associated with chloroquine-resistance (74I, 75E and 76T) were highly prevalent in all the groups, with a K76T mutation observed in 85% of isolates. Chemoprophylaxis with chloroquine (CQ) tended to be associated with an increased prevalence of the CVIET haplotype (i.e. 76% vs. 87% in isolates from patients without vs with chemoprophylaxis, NS). For *Pfdhfr-ts* 69% of the isolates were mutated, with only three mutations detected (N51I, C59R, N108S) (Fig. 2B). 36% of the isolates were triple mutants, 14% were double mutants and 17% were single mutants. Mutations were more prevalent in SM than MM (81% vs. 58%, OR 3.18 [0.6;18.3]) and also showed an higher rate of triple mutations (46% versus 31% of SM and MM respectively, OR NS).

### Large genetic diversity of the Dakar isolates and higher infection complexity in Cerebral Malaria

**Figure 3:**
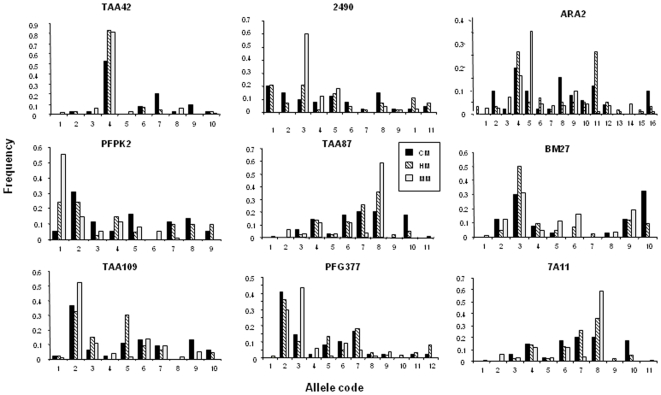
Allelic distribution of the nine microsatellites studied. Fragment size was calculated using a Rox-labelled internal molecular weight marker and cubic polynomial regression (Genscan software). For each microsatellite, alleles were defined according to the size of the PCR product and to the structure of the repeats in the genetic sequence. Size increases from code 1 to n. Frequencies were calculated in reference to the total number of samples in each group (CM: cerebral malaria, HM: hospitalized malaria, MM: mild malaria). Patient self-treatment was associated with higher frequency of ARA2 alleles 8-14; PFPK2 allele 2; TAA87 allele 7; and 7A11 alleles 1-2-4.

**Figure 4:**
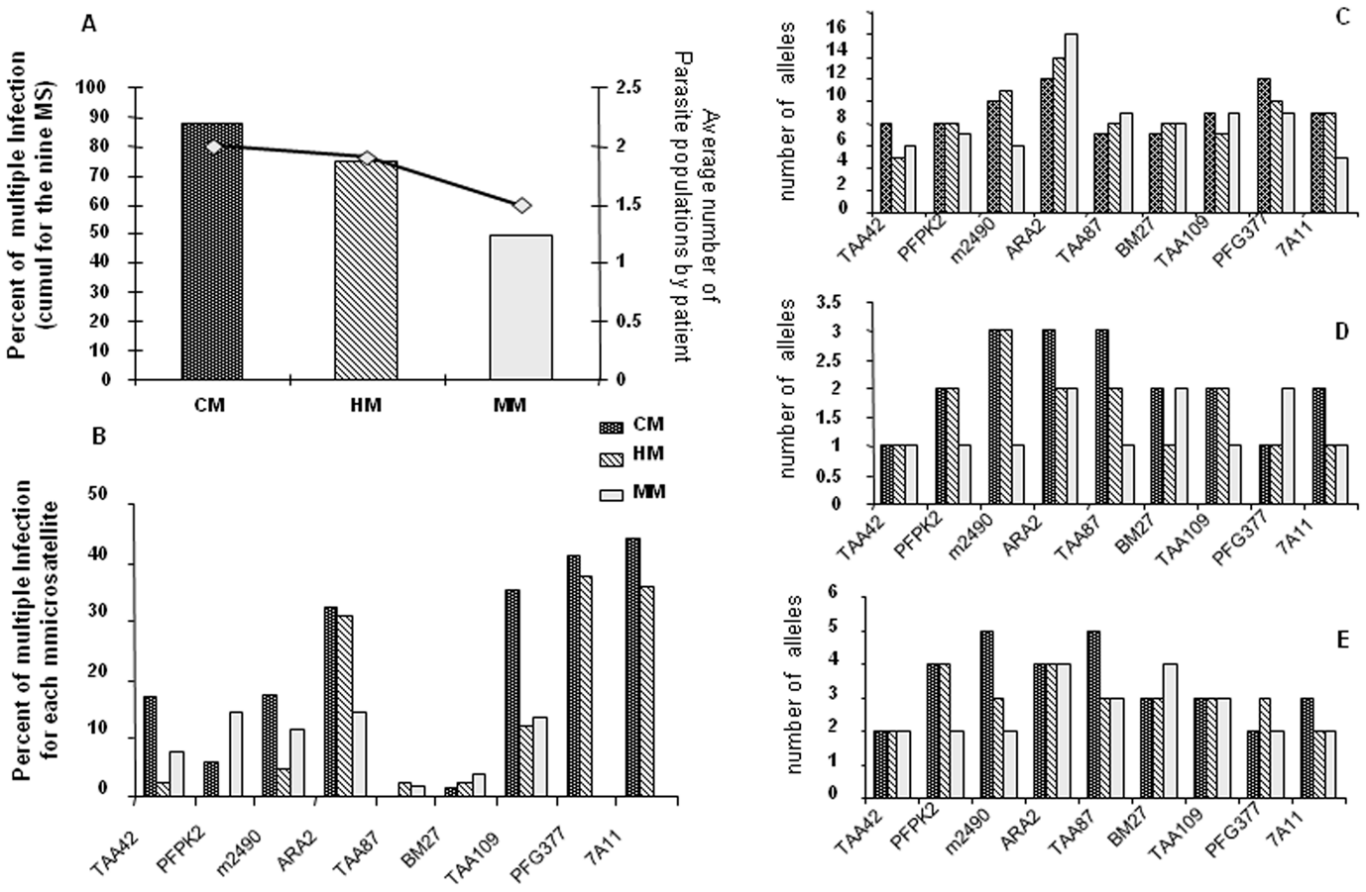
Genetic diversity of the parasite population. For a given patient group, the average number of alleles was calculated by the sum of distinct PCR fragments amplified from the group, divided by the number of persons in that group. A) Percentage of patients with multiple infections (deduced from 9-microsatellite loci genotypes) and number of alleles/patient by clinical group; CM were predominantly multi-infected (2.3 isolates/person). B) Percentage of patients with multiple alleles for each microsatellite in each clinical group, C) Total number of alleles for each microsatellite locus in each clinical group. ARA2, TAA109, PFG377, 7A11, TAA87 and BM27 presented a different infection complexity in CM, as compared to HM (Mann Whitney p = 0.0058, p = 0.013, p<0.00001, p<0.00001, p = 0.008, p = 0.008, respectively) D) Number of alleles common to 50% of the isolates in each clinical group. E) Number of alleles common to 75% of the isolates in each clinical group.

Despite the low transmission rate of malaria in Dakar, parasite isolates from the area contained highly polymorphic microsatellite loci, with 9 to 16 alleles observed per locus (Fig. 3). The total number of alleles per locus (Fig. 4C) was similar in SM and MM isolates (FSTAT analysis, NS) and was lower at CHN Fann due to the small sample size (data not shown, DNS). However, the frequency of alleles was variable, with a dominant allele for some loci (50% of the patients sharing the same 2 alleles and 75% the same three alleles, irrespectively of their clinical group, Fig. 4D–E), and a broad distribution for others. Six microsatellites presented a greater complexity in CM compared to HM and MM (Fig. 4B). However, no association of a specific allele was detected with any clinical group, possibly due to an insufficient sample number.

In contrast, self-treatment was associated with an increased frequency of some alleles, which could indicate a selection of parasites (DNS). However, no significant association between a specific allele and a *Pfcrt* or *Pfdhfr* haplotype was found (once again, this may be due to an insufficient sample number).

By taking the nine microsatellites together, it was found that the multiplicity of infection was high with a maximum of three distinct parasite populations detected per sample (Fig. 4A). It increased with the severity of the disease (KW, p = 0.001), with a 15-fold higher risk of CM in cases of multiple infection (OR CM/MM 15.3 [3.3;98], OR CM/HM 6.4 [1.2; 45]). Furthermore, all fatal cases were multi-infected (Mann Whitney with survivors p = 0.019). Surprisingly, multiplicity of infection was inversely correlated to the mean parasitaemia of the patients (Spearman R = −0.173, p = 0,029), but not to other clinical parameters or treatments. This is however consistent with lower parasitaemia detected in severe cases.

### Patients with severe malaria harboured a selected group of parasite isolates

**Figure 5:**
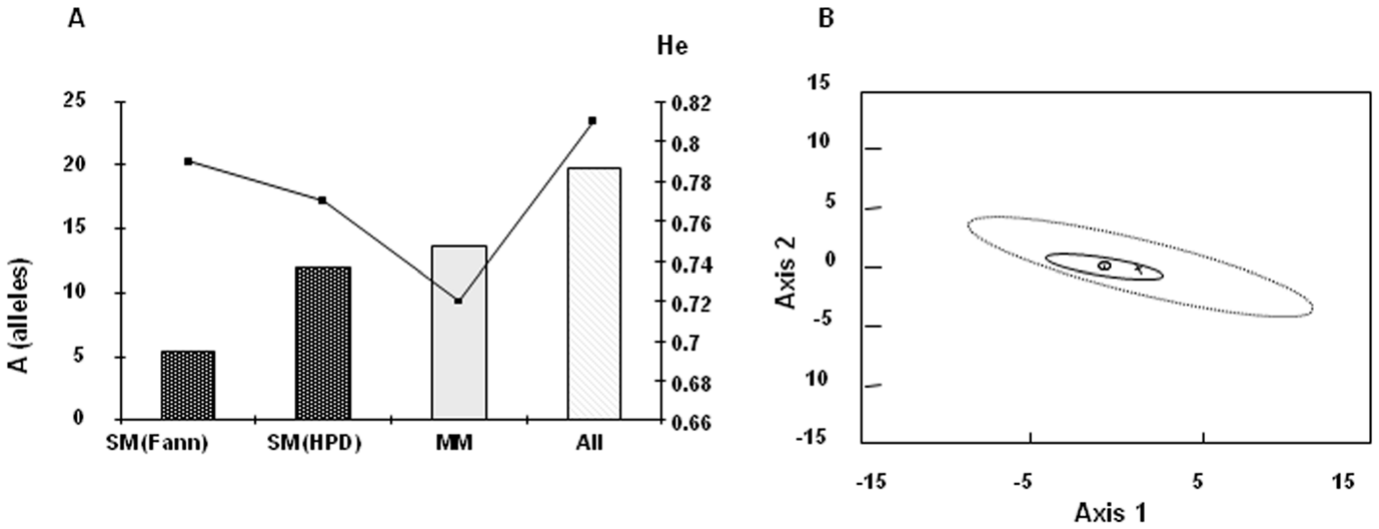
Genetic diversity of the parasite population (Fst). 193 genotypes were scored at 9 loci, 29 genotypes at 7 loci and 13 genotypes at 6 loci. Reconstruction of haplotypes was carried out for 235 genotypes (15, 77 and 143 at Fann, HPD and MM patients respectively) A) Mean number of distinct alleles observed per locus. Unbiased expected heterozygosity (He) per locus was calculated using the unbiased estimator of Nei corrected for small sample sizes, (SM) severe malaria  =  HM+CM. The total number of alleles per locus is similar in SM and MM isolates (FSTAT, A = 13.7±4.3 for HPD and 11.9±4.4 for dispensary, Fann A = 5.44±1.4). Parasite populations were genetically different for different clinical groups: Fst comparison CM (Fann)−MM = 0.160, Fst CM(HPD)-MM = 0.163, Fst Fann-HPD = 0.157 (p<0.017 for all). B) Canonical Correspondence Analysis of genotypes (CANOCO software) obtained from MM (n = 129 genotypes) and SM (HPD, n = 59 genotypes) at 9 microsatellite loci. The significance of the canonical axes was tested with 1000 Monte Carlo permutations with a 95% confidence interval of the centroid of each population (centroids of populations are surrounded by 95% confidence intervals: o and hashed trait ellipse =  MM; x and plain ellipse  =  HPD-recruited SM). CCA shows a larger genotypic diversity in SM isolates than in MM (Monte-Carlo, p-value = 0.002).

A high mean heterozygosity was found for the 235 haplotypes reconstructed (He = 0.81±0.07, Fig. 5A). Strong linkage disequilibrium was found between microsatellite loci. Linkage and FST analyses confirmed the selection of a specific group of isolates in SM. Indeed, for all isolates, 33 of 36 pair wise combinations of loci displayed significant linkage (p-value<0.0014), compared to 11/36 for SM and 4/36 for MM, respectively. This data supports evidence of a higher linkage of the parasite population in severe malaria compared to MM patients, and a low rate of exchange between these two parasite populations. Fst values also indicate a significant difference between these clinical groups (p<0.017 for all). Canonical Correspondence Analyses confirmed (Fig. 5B) a larger genotypic diversity in SM isolates than in MM, in line with the multiplicity of infection.

Overall, no significant linkage between SM and a specific parasite genotype/allele was detected by single microsatellite analysis. However, global genetic analyses outline a larger diversity of parasite populations in SM and a clear genetic difference between parasites associated with either SM or MM.

## Discussion

This study, conducted in Dakar during the peak of malaria transmission [6], [8], compares parasite isolates collected from patients attending one of the largest out-patient clinics in this area and from patients admitted to hospital for severe malaria with neurological impairment.

During this study, adult patients accounted for about 50% of all confirmed malaria cases at the dispensary. This high prevalence is unlikely to be due to under-representation of children, resulting e.g. from home treatment, as more than 70% of the consultations were for children <15 y. Adults were also highly susceptible to severe malaria, as they accounted for 87% of the 8% of the malaria cases referred to hospital, in agreement with other surveys at CHN-Fann [19], [20].

For the genotyping study, three groups of patients were compared: mild malaria, hospitalized malaria and cerebral malaria. A very high rate of chloroquine resistance-associated CVIET *Pfcrt*, haplotype was found in all clinical groups. Furthermore, a higher prevalence of mutated *Pf-dhfr* isolates was found in severe cases. These findings are compatible with other studies [21] showing elevated *in vitro* cycloguanil resistance in isolates collected at hospital. Self-treatment was reported by 16.8% of the MM patients attending the dispensary. This rate is lower than that previously reported by Ndiaye et al [22] and concerned mainly patients with HM (particularly men). Self-treatment associated with: i) a longer delay between appearance of symptoms and presentation at hospital ii) and a lower level of haemoglobin at admission. SP is still largely used by adults due to its single-shot administration. Presence of *Pfdhfr* mutants in SM may indeed reflect poor efficacy of this self-treatment. Additionally we found that chloroquine-prophylaxis tended to be associated with a higher frequency of a *Pfcrt* mutated genotype. Chloroquine and SP are thus poorly efficient treatments. As was reported recently, they can, however, reduce parasite densities and result in delay in malaria presentation, which can contribute to asthenia and anaemia and thus to referral to hospital [23]. A lower proportion of parasitaemia was found in severe malaria cases than in MM in contrast with previous studies conducted in this area, before the appearance of chloroquine and sulfadoxine-pyrimethamine resistance [24].

Microsatellite genotyping of the isolates showed a high prevalence of multiple infections with a higher infection complexity and a larger genetic diversity in parasite isolates from patients with CM and HM. These data are in agreement with Nzila [25] and Conway [26], but not with Ferreira [27]. All parasite isolates collected from patients who subsequently died were polyclonal. In a previous study in the same area [24], in line with Durand et al [28], we observed a lower frequency of multiple infections in severe cases as compared to mild cases, but a similar mean number of alleles in multiply infected patients from both groups. However, we used polymorphic genes coding for antigens potentially under immune selection rather than neutral microsatellites. This may have biased the estimate, as demonstrated in Dakar by Leclerc [29]. Only a minority of patients reported to have traveled outside the area of Dakar (19%) suggesting that most infections have been acquired locally, whatever their clinical group. Since malaria transmission in Dakar is low, the permanent resident parasite pool is expected to be limited. The large diversity observed, and the elevated infection complexity, support the hypothesis of an invasion of isolates from rural areas into the urban biotope. Indeed, as in most of the large African agglomerations, peri-urban suburbs are still connected to the countryside through intense population movement [30]–[32]. As a consequence, multi-infected parasite carriers coming from rural high malaria transmission areas [33]–[34] are likely to introduce parasites that are then secondarily propagated in the peri-urban farming settings. This provides a regular feed of parasites into of the urban biotope [35]. This may account for the elevated multiplicity of infection observed in the Dakar residents in our study and also in previous studies of hospitalized patients [21] and pregnant women [36]–[37].

Microsatellite genotyping showed roughly similar allele frequencies in CM, HM and MM. There was no association of any specific allele with a clinical group or fatal outcome, or between a specific microsatellite allele and *Pfcrt* or *Pfdhfr* haplotypes. However, Fst analysis and linkage disequilibrium confirmed differences between MM isolates collected from dispensaries and those from severe malaria, collected from referral hospitals. They suggest a non-panmictic structure in *P. falciparum* populations. As patients were matched on their household location and recruitment period, this cannot be due to different spatial origins of the samples, and isolation between MM and severe malaria is also unlikely to have occurred. The results indicate that severe malaria is not associated with a small subset of highly virulent genotypes/isolates, but rather demonstrates that it is associated with a highly polymorphic group of isolates presenting population characteristics different from parasites associated with mild malaria. These differences could be related either to drug resistance or to antigenic specificity. As self-medication and treatment delay were found to be major factors associated with SM, a strong involvement of drug resistance in the selection of a pool of isolates is suggested. However, this does not explain genetic isolation of these isolates from those inducing MM. This could be explained by their newly introduction into the urban biotope from other parts of the country, followed by their selection due to inappropriate treatment. However, the role of specific antigenic properties of these isolates in their pathogenicity cannot be ruled out; as the immune response developed by inhabitants of this low transmission area could be mostly strain-specific.

Whatever the origin of these isolates, this study highlights that young adults are the major group of patients attending dispensaries with mild malaria and hospitals with severe malaria in urban settings. According to the new strategy for the elimination of malaria proposed by WHO in countries with low transmission, this clinical profile could be the most prominent in the next 5 or 10 years in most of the African urban areas. National Malaria teams must be aware of this evolution to adapt their strategies. Multiple infections and highly mutated *Pf-dhfr* parasites were associated with severe malaria, but parasite isolates collected from severe malaria patients were clearly identified as a separate pool of parasites from those detected in MM. This supports the need for full sequence analysis of the genome of these parasites in order to understand their association with patho-physiology.
